# Pediatric renal lithiasis in Spain: research, diagnostic and therapeutic challenges, and perspectives

**DOI:** 10.3389/fped.2023.1294319

**Published:** 2023-12-07

**Authors:** Javier Lumbreras, Leire Madariaga, María Dolores Rodrigo

**Affiliations:** ^1^ Pediatric Nephrology Unit, Department of Pediatrics, Son Espases University Hospital, Balearic Islands Health Research Institute (IdISBa), Palma de Mallorca, Spain; ^2^ Pediatric Nephrology Department, Cruces University Hospital, IIS Biocruces Bizkaia, University of the Basque Country, CIBERER/CIBERDEM/EndoERN, Barakaldo, Spain

**Keywords:** kidney stone, renal lithiasis, registry, epidemiology, citrate, lumasiran

## Abstract

Incidence and prevalence of urolithiasis is apparently increasing worldwide, also among children and adolescents. Nevertheless, robust data have only been obtained in a few countries. In Spain, a voluntary Registry for Pediatric Renal Lithiasis has been active since 2015. Irregular participation limits its applicability, as well as its limitation to patients with a stone available for morphocompositional study, to obtain data about incidence and prevalence. On the other hand, findings about typology of stones and clinical and analytical characteristics of these subjects have been communicated in several meetings. Other valuable efforts in this field are the elaboration of guidelines for the collection and processing of urine samples for the study of urolithiasis in pediatric patients with the consensus of the Spanish Society for Pediatric Nephrology (AENP) as well as the Spanish Society for Laboratory Medicine (SEQC), the collaborative network RenalTube for the diagnosis of primary tubulopathies and the registry of patients with Primary Hyperoxaluria (OxalSpain). In many hospitals from the public healthcare system, pediatric nephrologists are the specialists in charge of the management of children with kidney stones, but there is no formal regulation on this competence. Other specialists, such as urologists, pediatric surgeons or pediatric urologists, in many cases do not offer a complete insight into the etiopathogenic mechanisms and the consequent medical treatment. Access to medication according to standards of treatment is warranted, provided a correct diagnosis is achieved, but criteria for the reimbursement of certain therapies, such as RNAi drugs for primary hyperoxaluria, are arguable.

## Introduction

Incidence and prevalence of renal lithiasis has markedly increased in the last decades in adults, whether abroad or in Spain (1–3). Nevertheless, epidemiologic data are not homogeneous across regions, showing relevant differences which could relate to genetic, environmental or behavioral (including dietetic) factors. Data regarding pediatric patients are more limited, as it is only in Iceland and the USA that large studies have been performed. Nevertheless, a remarkable increase has also been observed there (4, 5). However, clinicians perceive that they must deal with this condition more frequently in the last years. Several initiatives to increase our knowledge about this condition as well as diagnostic skills and tools have been implemented in the last years. Therapeutic issues will also be discussed in this article.

## Epidemiology and trends of pediatric renal lithiasis in Spain

Prevalence of renal lithiasis in adults has been described as high as 16% in Andalusia (Spain) (3) or 8.8% in the United States of America in the period 2007–2010 (2), with an increase up to 11% (95% CI 10.1–12) in the period 2015–2018, according to the NHANES study (6). A 54% (95% CI 49.2–58.5) were men (6). Although data from previous decades are missing for the whole of Spain, there has been a dramatic increase in other countries (3.2% in 1980 in the USA, for example), which can be probably extrapolated to our population. Higher prevalence has been observed in individuals with obesity or diabetes, suggesting behavioral and environmental factors behind these changes (2).

Pediatric studies in renal lithiasis regarding incidence or prevalence are scarce but well documented. In Iceland, the mean annual incidence for population under 18 years old for the period 1999–2013 was 48/100,000 individuals and year for boys and 52/100,000 for girls. Annual incidence rose from 3.7/100,000 1985–1989 to a maximum of 11/100,000 in 1999–2005, then declining to 8.7/100,000 in the period 2010–2013 (4). This increase was mostly related to a dramatic rising in incidence among female adolescents (9.8/100,000 at the beginning of the study, 39.2/100,000 at the end) (4). In the USA, mean annual incidence in the period 2005–2016 has been estimated in 59/100,000 people under 18 years old, but with a top 65/100,000 in the last year of the study (5). A higher incidence in female adolescents was also observed in this study (5).

In the global population, calcium oxalate is the most common species in kidney stones. Calcium phosphate is also commonly found in a variable proportion of the composition. In many countries, calcium oxalate monohydrate is the most common type of stone (considering it as the main component) (7). Uric acid stones are relatively more prevalent in developing than in industrialized countries (8). Regarding specifically the pediatric population, in the Icelandic study, all the calcium oxalate stones accounted for the 60% of the cases in which it had been analyzed, and struvite only for a 10% (4). A monocentric study from the Great Ormond Street Hospital (GOSH) in London (UK) showed a 34% of calcium oxalate stones, 44% of hydroxyapatite ones, and 13% of struvite (9).

In Spain, no comprehensive or regional data are available in pediatric population, to our knowledge. An indirect marker for the burden of the disease, the rate of admissions related to renal colic, has been suggested to be between 1 and 5/1,000 children under 15 years of age per year in two Spanish hospitals for the years 2015–2017 (10). It has been estimated in 1/685 children under 18 years of age in the USA and 2.5/1,000 in Croatia (11). In the Balearic Islands, we have the opportunity to estimate in a relatively reliable way the epidemiologic markers of the disease in the pediatric age as most of patients are referred to a single pediatric nephrology unit. In the period 2008–2018, a mean annual incidence of 2.5 patients under 18 years of age (1.1 cases/100,000 people under 18 years of age and year) were diagnosed with stone available for study (10), 10 out of the 25 patients in the first four years of the study. Extrapolating the incidence in Iceland to the population under 18 years old in the Balearic Islands in 2013 (223,286 inhabitants under 18 years of age), local incidence should have been about 20 cases per year. Even if we are only comparing cases with available stone, we could assume that incidence would not be as high as it is in Iceland. On the other hand, over a period of 18 months between 2021 and 2022, a total of 130 patients (prevalent and incident cases) were treated at the outpatient clinic with conditions from the spectrum of renal lithiasis (53 of them with nephrocalcinosis or prelithiasic manifestations: hematuria, cloudy urine, chronic abdominal pain, recurrent dysuria, recurrent urinary tract infections or overactive bladder associated through clinical and metabolic workup with urine crystallization). Considering that this outpatient clinic receives a total of 1,200 visits per year (including first and follow-up visits), patients with renal lithiasis and related conditions represent a significant part of the workload (personal communication).

## National strategies to improve knowledge in pediatric renal lithiasis

In order to collect more information about renal lithiasis in Spanish children and adolescents, and to relate morphocompositional results from the study of kidney stones with clinical and analytical data, a national registry for patients under 18 years old was promoted by the Spanish Society for Pediatric Nephrology (AENP). Data collection started in 2015. Kidney stones included in this study are analyzed by a single specialized laboratory. Volunteer participation as well as bureaucratic issues have limited inclusion of patients, so that it cannot be applied to obtain reliable data about incidence or prevalence. Nevertheless, descriptive and analytical data regarding typology and its relationship with age and other factors have been obtained. Data from an interim analysis with data until the end of 2018 were presented in the International Pediatric Nephrology Association meeting in 2019. Eighty-four stones from 69 patients had been studied. Seventy-nine percent of them appeared in males. Diagnosis had been made at a median age of 6.5 years (IQR 3.1–12.7). First-degree relatives with kidney stones appeared in the 42% of the cases. It is remarkable that only in 51% of the cases, the typical presentation with acute abdominal pain was observed, probably due to the high proportion of patients with a short age when a casual finding or unspecific symptoms are more common. The more commonly observed types of stones were: calcium oxalate dihydrate (32.5%), struvite (19.3%), hydroxyapatite (13.3%) and calcium oxalate monohydrate from cavities (9.6%). The most prevalent metabolic alterations were: hypercalciuria (43.5%), hypocitraturia (28.6%), hyperoxaluria (17%). Calcium/citrate ratio >0.33 mg/mg was found in 44.6%. When dividing the sample in stones from patients under or above 6 years of age, statistically significant differences were observed in the following variables: positive family history for lithiasis much more common in the oldest ones (53.8% vs. 91.2%); on the contrary, past urinary infection was much more common among the youngest (56.7% vs. 14.3%), what was in concordance with the fact that CAKUT or dysfunctional bladder appeared in 31% vs. 8.6%, respectively. Spontaneous elimination was attained in 50% of the youngest but in 80% of the oldest ones. Diagnosis was more commonly a casual finding under 6 years of age (38.5% vs. 11.4%). Regarding metabolic evaluation of the patients, several variables were close to statistical significance, such as calcium to citrate ratio 0.26 [0.11–0.70] vs. 0.43 [0.18–1.16] mg/mg, higher in the group above 6 years of age. Calcium oxalate dihydrate stones were found less frequently among the youngest patients (13.3% vs. 45.7%), but struvite was practically only observed under 6 years of age (26.7% vs. 2.9%), which is consequent with the aforementioned clinical and biochemical characteristics of both groups. That is to say, even if metabolic factors were common at all ages, urinary infections and structural or functional abnormalities of the urinary tract played a role under 6 years of age, but not beyond (12). The typology of the stones was the one from developed countries (8). Nevertheless, regional differences are observed comparing this study with previous ones. Calcium oxalate stones (even taken as a whole) were less frequent than in the Icelandic study but with a similar proportion to the study form the GOSH. The proportion of hydroxyapatite stones was smaller than in the British study (which showed a remarkably high prevalence). Finally, the frequency of struvite stones was higher in the Spanish registry than in the both aforementioned studies (4, 9). Uric acid stones show a low frequency in the three studies. The morphocompositional classification in the Spanish registry is more specific in terms of details, for example regarding calcium oxalate stones. The classification proposed by Grases et al. is used for this purpose (13). The registry is still open and a reanalysis is foreseen.

Other initiatives to improve the knowledge about specific conditions related to lithiasis are OxalSpain and RenalTube. OxalSpain is a national registry for patients with Primary Hyperoxaluria (PH). It can ease contact with a specialized lab for diagnostic workup and collects clinical and analytical data from adult and pediatric patients. It aims at giving visibility to this disease to improve resources for treatment, as well as improve understanding of the disease.

RenalTube is a national collaborative project with a network-based registry that collects clinical data of patients with 23 different primary tubulopathies, offering in exchange specific genetic diagnosis for these patients (14). Some of these conditions are very frequently associated with nephrocalcinosis or renal lithiasis, namely Bartter syndrome, familial hypomagnesemia with hypercalciuria and nephrocalcinosis, distal renal tubular acidosis, Dent disease, and hypophosphatemic rickets.

Apart from these initiatives, certain centers are collaborating with other transnational European initiatives. One of them is OxalEurope (www.oxaleurope.org), a consortium with the objective of improving detection and treatment of hyperoxaluria across Europe. Another one is ERKNet (www.erknet.org), another consortium focused on rare kidney diseases that supports ERKReg, a registry for rare kidney diseases including some of them being causative for monogenic renal lithiasis. Specifically, subregistries have been settled for distal renal tubular acidosis, cystinuria and Bartter disease.

## Diagnostic procedures: opportunities to improve clinical and laboratory practices

An adequate collection and processing of the urine samples are essential for the diagnostic workup in renal lithiasis. Nevertheless, which the best sample is, how many samples must be collected and when, are still controversial topics (15–18). Certain efforts have been made to achieve a higher consensus in this field (19). Besides, methodology followed when collecting and processing urine is variable among centers, and not always attached to the present recommendations. At least part of these recommendations is frequently not based on a high level of evidence (19).

In a national survey conducted in 2017 among pediatric nephrologists and promoted by the AENP, we observed several improvement opportunities. Results were presented in the yearly meeting of the society. A total of 26 responses from 25 centers were obtained. Only 64% had a protocol about urine collection for the metabolic workup in renal lithiasis. The type of urine sample (24 h, 12 h, spot sample) in which the parameters were measured was variable. Postprandial sample to evaluate effect of oral intake on calciuria was not commonly performed. pH was determined exclusively with dipsticks in 84% of the centers, when real pH can vary up to 0.5 above or below, which is relevant in certain cases such as in the diagnosis and management of patients with renal tubular acidosis or with cystinuria, or in general with the adjustment of alkali (citrate or bicarbonate) therapy. Only 28% of the responders could rely on its own laboratory; in the rest of the centers, part of the parameters had to be determined in other laboratories, thus being compelled to send urines with the consequent risk of preanalytical interferences. In certain cases, no measures (addition of thymol or refrigeration during collection) were taken to avoid bacterial overgrowth in 24 h or 12 h samples, with the risk of biasing in certain parameters such as pH or citraturia (20).

AENP decided to contact with the Spanish Society for Laboratory Medicine (SEQC^ML^) to create a conjoint working group and elaborate a consensus statement about procedures for the metabolic workup in pediatric renal lithiasis, including preanalytical and analytical considerations. The working group reviewed the literature and found that the level of evidence in this field was not enough to give graded recommendations. Previous recommendations from the European Association of Urology and the American Urology Association, and other societies, had been published but not from a specific pediatric point of view, even with certain considerations for children and adolescents (21–23). Specific guidelines for certain diseases have been also published. One example is the clinical recommendations published by OxalEurope-ERKNet in 2023 for the diagnosis and management of Primary Hyperoxaluria (24). Other recommendations can be found but not with the category of guidelines (18, 25). In general, in the literature, exhaustive metabolic workup is not universally recommended in adults although suggested in children. Preanalytical and analytical issues for each solute and pH are not addressed in a single document. A document based on the existing bibliography with recommendations based on the experts' interpretation of the reviewed articles was written and published (26). The main recommendations can be summarized in obtaining multiple samples (diagnosis cannot be based on a single one), avoiding bacterial overgrowth during collection, and the need to acidify urine for the measurement of oxalate and its related metabolites (but not necessarily during the collection). The exact number of samples and the possibility of studying periods shorter than 24 h or using spot samples is discussed remarking their benefits and limitations (26).

Even if it was not the main objective of the consensus statement, considerations about processing stones were also included. Access to a proper study of kidney stones is still irregular, considering present recommendations (7, 19). Biochemical analysis is known to be associated to an unacceptable rate of inaccurate results (19) but it is still performed elsewhere. Only stereomicroscopy, Fourier-transformed infrared spectroscopy or x-ray diffraction, or even better a combination of them, are acceptable techniques for the morphocompositional study of kidney stones (7, 13). The study of kidney stones from a combined structural and biochemical point of view can give significant information on its formation and evolution under consequent therapies, thus influencing diagnosis, treatment, prognosis of the disease as well as preemptive measurements.

This consensus has been specifically elaborated for pediatric patients. Even if many advises can be applied to adult patients regarding preanalytical and analytical issues, a complete applicability is not warranted.

## Management of pediatric renal lithiasis

In Spanish pediatric patients, although acute management of kidney stones is usually performed by pediatric or adult urologists, it is the role of the pediatric nephrologist to carry out a metabolic study to evaluate any underlying pathology or metabolic condition that increases the risk of recurrent lithiasis.

As in adults, general preventive measures to avoid recurrent stones are mandatory in children. These include increasing urinary output, avoiding excess oxalate and animal protein intake, and salt restriction (27). Children with idiopathic hypercalciuria and recurrent lithiasis are usually treated with potassium citrate to reduce the available ionic calcium and avoid crystal aggregation, or a thiazide diuretic to increase tubular calcium reabsorption. In those cases where hypocitraturia is the leading defect, treatment with potassium citrate is mandatory (28). In Spain, potassium citrate is available and reimbursed by the public health system only in the solid or liquid forms. Potassium citrate powder is licensed as a nutritional supplement instead of a drug, which hinders its reimbursement. This complicates the treatment of hypocitraturia in children who are too young to swallow tablets (potassium citrate tablets are extended release and cannot be split) but too old to take liquid forms (because of the high volume due to low concentration of the solutions).

In pediatric patients with primary tubulopathies, nephrocalcinosis and/or lithiasis are frequent complications, usually associated with hypercalciuria and/or hypocitraturia, which are, to some extent, secondary to the electrolytic defects. Nephrocalcinosis has been implicated as a risk factor for the development of CKD over time in some primary tubulopathies, and efforts are made to revert this situation. In this way, diuretic thiazides are frequently used in our country to treat hypercalciuria and nephrocalcinosis in patients with Dent disease or Familial hypomagnesemia with hypercalciuria and nephrocalcinosis. Furthermore, a large survey within Europe in patients with Dent disease reported the use of thiazides in 35% of these patients (29). However, the success of this treatment in reverting nephrocalcinosis or in decreasing the rate or speed of the development of CKD is questionable (30). Some experts also suggest that volume depletion induced by thiazides in pathologies that frequently associate polyuria, may worsen renal function prognosis over time despite achieving a decrease in calcium excretion and in the incidence of renal stones (31). Finally, renal lithiasis in children with primary tubulopathies is a risk factor for recurrent acute pyelonephritis and/or obstructive episodes, which may in turn increase the risk of CKD over time. Prompt evaluation and treatment of obstructive lithiasis in these children is mandatory. In our country, the management of these children is usually done in tertiary referral hospitals with pediatric urologists.

Recently, a new prolonged-release formulation of potassium citrate and potassium bicarbonate (ADV7103), approved for the treatment of patients with distal renal tubular acidosis above 1 year of age, has shown to improve metabolic control in these patients (32). In addition, gastrointestinal tolerability was also shown to be better than with the traditional bicarbonate and citrate supplements (33). This better metabolic control could decrease the risk of nephrocalcinosis or lithiasis and its related complications, although this has not been demonstrated yet. Distal renal tubular acidosis progresses to CKD in a significant number of cases over time, with nephrocalcinosis as a significant and very frequent complication (34, 35). Despite the results shown, at present ADV7103 is not reimbursed by the public health system in our country and patients with this disease are generally treated with the classical bicarbonate and citrate approach.

Phytate is present in significant amounts in food such as cereals, nuts and beans. It can help chelating calcium in the intestinal lumen as well as in the tubules (11, 36). Nevertheless, it is not widely clinically use and specific dosage and presentations for pediatric patients are lacking. It can only be quantified in urine samples in research laboratories.

Finally, Primary Hyperoxaluria (PH) is a hereditary disorder, involving the overproduction of oxalate. PH type 1, the most common form, is caused by a deficiency of the liver peroxisomal enzyme alanine-glyoxylate-aminotransferase (AGT), encoded by the *AGXT* gene. This enzyme catalyzes the conversion of glyoxylate to glycine. When AGT activity is absent, glyoxylate is converted to oxalate, which forms insoluble calcium oxalate crystals that accumulate in the kidney and other organs. Hyperoxaluria leads to nephrocalcinosis and/or renal lithiasis, and development of CKD in many cases in the first decades of life (37). Potassium citrate has demonstrated in patients with PH to significantly decrease calcium oxalate saturation, although the ability of this treatment to reduce the rate of kidney stones or nephrolithiasis, or even the long-term rate of CKD is unclear (38). This also holds true for patients with secondary hyperoxaluria due to an increased intestinal oxalate reabsorption (39). Recently, lumasiran, an RNA interference (RNAi) therapeutic agent that reduces hepatic oxalate production by targeting glycolate oxidase, has been approved by the Food and Drug Administration (FDA) and the European Medicines Agency (EMA) for the treatment of PH (40). Lumasiran is administered subcutaneously, and it specifically targets hepatocytes to silence the *HAO1* gene encoding the glycolate oxidase. This reduces the amount of glyoxylate which is a substrate for oxalate production. Since the glycolate oxidase enzyme is upstream of the deficient AGT, that causes PH type 1, the mechanism of action in lumasiran is independent of the underlying *AGXT* gene mutation. However, lumasiran is not expected to reduce hepatic oxalate production to the same extent in patients with PH type 2 and 3 (41, 42). Lumasiran has shown to significantly decrease the levels of oxalate in urine and plasma, compared to placebo (41). In addition, Lumasiran decreases the rate of nephrocalcinosis in these patients (43). This will probably improve the renal prognosis in these patients over time, Recently, Nedosiran, another RNA interference (RNAi) therapeutic agent, has been approved by the FDA for children with PH type 1. Nedosiran inhibits the expression of the hepatic lactate dehydrogenase (LDH), encoded by the *LDHA* gene. This enzyme is thought to be responsible for the terminal step of oxalate synthesis. Nedosiran could also be effective in patients with PH type 2 and 3, although results are inconclusive in these groups (44, 45). In Spain, lumasiran is indicated (and financed) in children 2 years or older, with genetic confirmation of the disease, glomerular filtration rate >45 ml/min/1.73 m^2^, and no evidence of severe hepatic or extrarenal disease (Therapeutic Positioning Report PT/V1/68/2022). However, children diagnosed early in life may develop severe CKD before the age of 2 years, so that being excluded from this new promising therapy.

## Conclusions and perspectives

Different strategies have been developed in our country to improve our knowledge about the nationwide epidemiology of lithiasis in pediatric population as well as in specific populations (primary renal tubulopathies and primary hyperoxaluria). A consensus statement has been published addressing the metabolic workup in the diagnostic approach of these patients. Commitment from pediatric nephrologists in the diagnosis, treatment and research in renal lithiasis plays a crucial role for an adequate management of the patients. New molecules have been developed in the last years to treat specific diseases, although reimbursement is controversially limited to certain cases.

## Data Availability

The original contributions presented in the study are included in the article/Supplementary Material, further inquiries can be directed to the corresponding author.
